# Prevalence of nocturnal hypoglycemia in free-living conditions in adults with type 1 diabetes: What is the impact of daily physical activity?

**DOI:** 10.3389/fendo.2022.953879

**Published:** 2022-09-27

**Authors:** Joséphine Molveau, Rémi Rabasa-Lhoret, Étienne Myette-Côté, Virginie Messier, Corinne Suppère, Kathryn J. Potter, Elsa Heyman, Sémah Tagougui

**Affiliations:** ^1^ Institut de recherches cliniques de Montréal, Montréal, QC, Canada; ^2^ Département de Nutrition, Faculté de Médecine, Université de Montréal, Montréal, QC, Canada; ^3^ Univ. Lille, Univ. Artois, Univ. Littoral Côte d’Opale, ULR 7369 - URePSSS - Unité de Recherche Pluridisciplinaire Sport Santé Société, Lille, France; ^4^ Département des Sciences Biomédicales, Faculté de Médecine, Université de Montréal, Montreal, QC, Canada; ^5^ Endocrinology Division, Montreal Diabetes Research Center, Montréal, QC, Canada; ^6^ Department of Applied Human Sciences, Faculty of Science, University of Prince Edward Island, Charlottetown, PE, Canada; ^7^ Institut Universitaire de France (IUF), Paris, France

**Keywords:** type 1 diabetes, nocturnal glucose control, hypoglycemia, physical activity level, accelerometer, continous glucose monitoring

## Abstract

**Objective:**

Studies investigating strategies to limit the risk of nocturnal hypoglycemia associated with physical activity (PA) are scarce and have been conducted in standardized, controlled conditions in people with type 1 diabetes (T1D). This study sought to investigate the effect of daily PA level on nocturnal glucose management in free-living conditions while taking into consideration reported mitigation strategies to limit the risk of nocturnal hyoglycemia in people with T1D.

**Methods:**

Data from 25 adults (10 males, 15 females, HbA_1c_: 7.6 ± 0.8%), 20-60 years old, living with T1D, were collected. One week of continuous glucose monitoring and PA (assessed using an accelerometer) were collected in free-living conditions. Nocturnal glucose values (midnight–6:00 am) following an active day “ACT” and a less active day “L-ACT” were analyzed to assess the time spent within the different glycemic target zones (<3.9 mmol/L; 3.9 – 10.0 mmol/L and >10.0 mmol/L) between conditions. Self-reported data about mitigation strategies applied to reduce the risk of nocturnal hypoglycemia was also analyzed.

**Results:**

Only 44% of participants reported applying a carbohydrate- or insulin-based strategy to limit the risk of nocturnal hypoglycemia on ACT day. Nocturnal hypoglycemia occurrences were comparable on ACT night versus on L-ACT night. Additional post-meal carbohydrate intake was higher on evenings following ACT (27.7 ± 15.6 g, ACT vs. 19.5 ± 11.0 g, L-ACT; P=0.045), but was frequently associated with an insulin bolus (70% of participants). Nocturnal hypoglycemia the night following ACT occurred mostly in people who administrated an additional insulin bolus before midnight (3 out of 5 participants with nocturnal hypoglycemia).

**Conclusions:**

Although people with T1D seem to be aware of the increased risk of nocturnal hypoglycemia associated with PA, the risk associated with additional insulin boluses may not be as clear. Most participants did not report using compensation strategies to reduce the risk of PA related late-onset hypoglycemia which may be because they did not consider habitual PA as something requiring treatment adjustments.

## Introduction

Type 1 diabetes (T1D) is a chronic condition caused by the autoimmune destruction of pancreatic beta cells, eventually resulting in absolute insulin deficiency, leading to hyperglycemia (1). Thus, people living with T1D require life-long insulin replacement therapy with the goal of maintaining glucose levels close to normal while minimizing the risk of iatrogenic (i.e. complication induced by the treatment) hypoglycemia (blood glucose [BG] < 3.9 mmol/L) (1). Despite therapies (e.g. insulin analogs) and new technologies (e.g. continuous glucose monitoring (CGM)), the risk of hypoglycemia remains high, especially at night (2). Mild to moderate hypoglycemic episodes ([BG] between 3.9 – 3.0 mmol/L) commonly occur during the night (3–5), and can last for over an hour in people living with T1D (6). More than half of severe hypoglycemic episodes (i.e. requiring someone’s assistance for recovery) occur during sleep (7). Thus, people with T1D are often still challenged with nocturnal hypoglycemia in their everyday life (8). Several factors in people’s daily life, such as bedtime BG level, daytime hypoglycemia and physical activity (PA) have been associated with an increased risk of nocturnal hypoglycemia (9). PA results in significant glucose fluctuations during and after exercise, especially hypoglycemia. Hypoglycemia may occur during, immediately after, and for up to 31-h after PA (10–12). People with T1D are unable to reduce circulating insulin levels without anticipation. In addition to an increased insulin sensitivity in the hours following PA (13), counterregulatory hormone response to glucose lowering is frequently altered in people with T1D (14). Increased insulin sensitivity associated with excessive circulating insulin levels and a frequently altered hormonal counter-regulatory response to hypoglycemia, predispose to nocturnal hypoglycemia, especially when PA is involved late in the afternoon (15–17). Repeated episodes of hypoglycemia may impair hypoglycemia awareness and thus further potentiate the risk of recurrent hypoglycemia (18, 19). Impaired awareness of hypoglycemia in people living with T1D can be fatal (18).

Though regular PA is highly recommended for its numerous health benefits such as improved physical fitness and cardiovascular health (20–22), many people with T1D fail to meet the national PA guidelines (23). The most commonly reported barrier to PA in people living with T1D is the fear of hypoglycemia (24).

Although strategies exist to mitigate the risk of hypoglycemia during and after PA, most studies evaluating these strategies are conducted in standardized, controlled conditions (25–30). Moreover, limited evidence-based data are available on delayed-onset hypoglycemia and very few studies have evaluated mitigation strategies to reduce the risk of PA-associated nocturnal hypoglycemia (31–36). Current guidelines recommend a 20% insulin basal rate reduction around bedtime, for 6 hours for people using continuous subcutaneous insulin infusion (CSII) (37). Evening snacks are an option as well, but evidence supporting this strategy remains mitigated (34). It is unclear whether people with T1D apply some of these strategies or not. Spontaneous PA often results in an accumulation of short bouts of PA throughout the day which can be an easier way for people to meet PA guidelines (38). However, for people living with T1D, identifying whether therapeutic adjustments (such as insulin reduction or carbohydrate (CHO) intake) are needed when PA occurs sporadically throughout the day may be more difficult, especially since the risk of nocturnal hypoglycemia associated with an accumulation of short bouts of PA through the day is not well known. Few studies have shown an increased risk of prolonged nocturnal hypoglycemia with the accumulation of moderate or vigorous intensity PA through the day (12, 39).

PA-associated nocturnal hypoglycemia remains a substantial clinical problem for T1D management, and further research in non-standardized conditions is required to overcome it. The objective of this study is to assess the effect of PA on nocturnal glycemic fluctuations in people living with T1D in free-living conditions, by taking into consideration reported mitigation strategies to limit the risk nocturnal hypoglycemia.

## Research design and methods

We carried out a cross-sectional, descriptive study to collect information about PA and glucose management in people with T1D during a usual week. Fifty-eight adults living with T1D were enrolled at the Montreal Clinical Research Institute (IRCM). The present study was approved by the research ethics committee and carried out in accordance with the principles of the declaration of Helsinki.

Inclusion criteria included having a diagnosis of T1D for at least 6 months, age ≥18 years, use of CSII or multiple daily injections (MDI), and the ability to give informed consent. Exclusion criteria included abnormal blood panel and/or anemia, ongoing or planned pregnancy, and impaired decision-making capacity.

## Study procedures

Participants were tested at the IRCM during two visits scheduled approximately 1 week apart. During the first visit, an accelerometer (SenseWear Armband Mini^®^ from Bodymedia.) was placed on the participant’s right arm and a blinded CGM (iPro™2 professional from Medtronic) was inserted subcutaneously on the opposite arm. Participants were asked to measure capillary blood glucose levels at least four times per day, using their own glucose meter for subsequent CGM sensor calibration. Participants wore the CGM and accelerometer for 6 days following visit 1. Participants were asked to complete a logbook every day during the 6 days they were wearing the CGMs and accelerometer to report their capillary blood glucose values, any hypoglycemic events (with or without symptoms and means of correction) as well as any relevant information regarding their insulin administration (i.e., insulin boluses, insulin reduction etc…). Glycated hemoglobin (HbA_1c_) levels were obtained *via* veinous sampling during visit 1. Hypoglycemia awareness was measured using the Clarke questionnaire. A score ≥ 4 indicates impaired awareness of hypoglycemia; a score ≤ 2 indicates normal awareness of hypoglycemia; and a score of 3 indicates undetermined awareness status (40, 41).

### Identification of active and less active days through objective measurement of PA

The Sensewear Armband includes a two-axis accelerometer and uses sensors to measure heat flux, galvanic skin response, skin temperature and near body ambient temperature to assess energy expenditure. It has been validated to measure daily expenditure in previous studies (42, 43). Data was downloaded on the Sensewear Professional Softwear. PA was divided into four categories: light (1.6 - 3.0 Metabolic equivalent of Task (METs)), moderate (3.0 - 6.0 METs), vigorous (6.0 – 9 METs) and very vigorous (≥ 9 METs) (44). Based on the downloaded data, the software then calculated time spent in different PA intensities each day.

### Identification of active and less active days

Our data analysis was based on PA level (defined as energy expenditure divided by basal metabolic rate in 24h). PA score between 1.40 and 1.69 was associated with a sedentary or light activity lifestyle, between 1.70 and 1.99 with an active or moderately active lifestyle, and between 2.0 and 2.40 with a vigorous or vigorously active lifestyle as defined (45, 46). Thus an active day (ACT) was defined as a PA level ≥ 1.7 and a less active day (L-ACT) was defined as PA level ≤ 1.69.

Some data cleaning procedures were required to perform our statistical anlaysis:

-If the participants’ data did not include at least one sedentary day (PA level ≤ 1.69) and one active day (PA level ≥ 1.7), the data was rejected from the analysis.
-If data from the CGM and/or accelerometer were unreadable or corrupted, they were excluded as well.

### Nocturnal hypoglycemia through CGM

The CGMs were blinded to the participant. Data was downloaded by our team on Carelink after the second visit.

CGMs were calibrated retroactively using the participants’ daily capillary glucose values reported in their logbook.

Level 1 hypoglycemia was defined as glucose levels between 3.0 and 3.9 mmol/L. Level 2 hypoglycemia was defined as glucose levels below 3.0 mmol/L. Level 1 hyperglycemia was defined as glucose levels above 10.0 mmol/L and level 2 hyperglycemia was defined as glucose levels >13.9mmol/L (47). Coefficient of variation (CV), defined as the standard deviation divided by the mean was calculated as well. CV < 36% indicated stable glucose levels (47).

**Figure 1:**
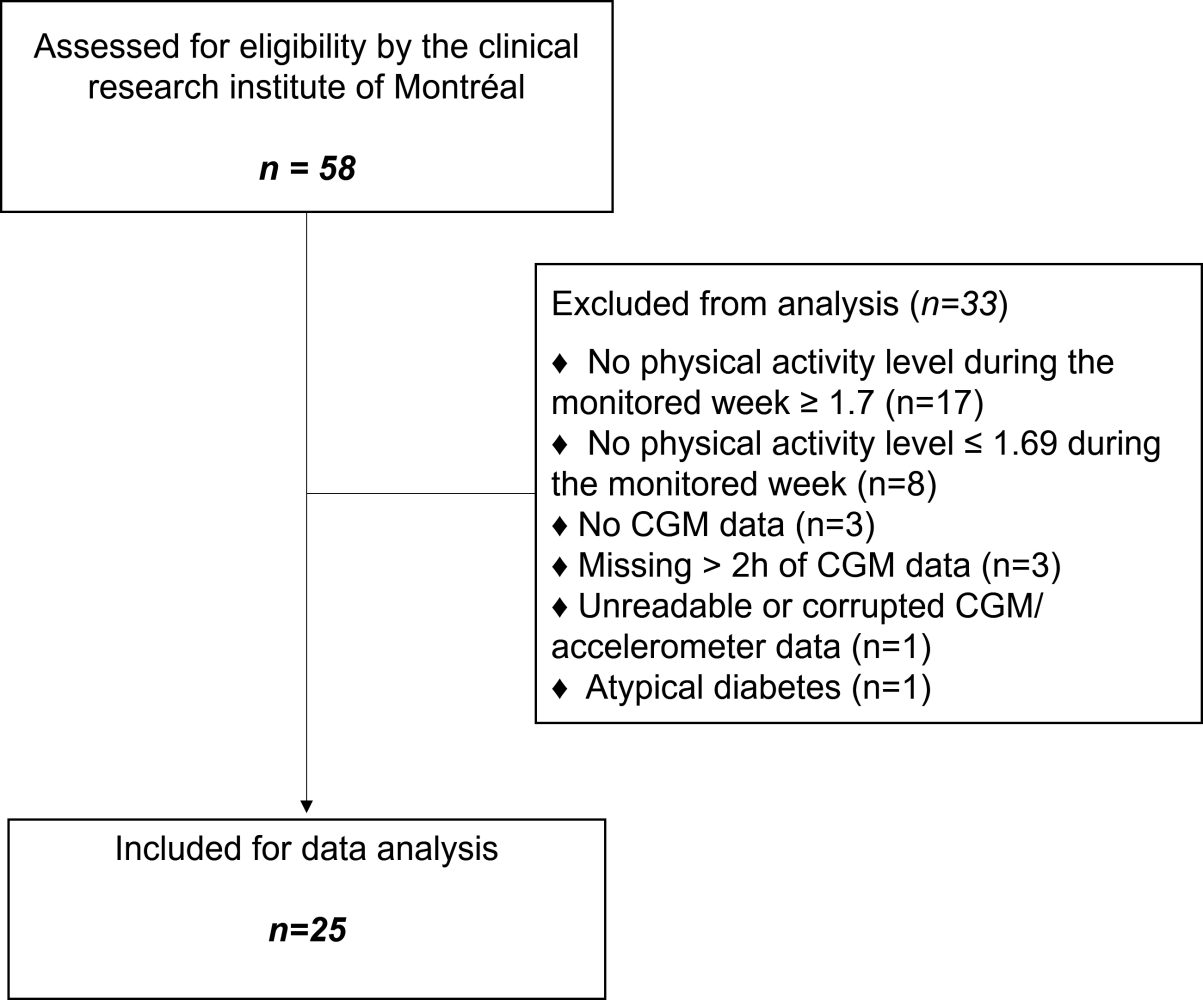
Consort flow diagram of the study.

Nocturnal glucose levels were analyzed from 00:00 (midnight) to 6:00 am. Nocturnal hypoglycemia was defined as BG < 3.9 mmol/L for at least 15 consecutive minutes. If more than two hours of CGM values were missing between 00:00 am and 6:00 am, the data was rejected from the analysis (**Figure 1**).

### Mitigations strategies reported in the logbook

Information about PA (time of day, duration, and type), hypoglycemia occurring before bedtime, and mitigation strategies (*e.g.* insulin dosage and CHO intake modulations) to reduce the risk of nocturnal hypoglycemia were based on the participants’ self-reported data in their logbook. Logbook information about mitigation strategies applied before bedtime was analyzed for ACT and L-ACT days only.

### Statistical analysis

We compared nocturnal blood glucose levels after ACT with nocturnal blood glucose levels after L-ACT based on CGM data. Descriptive analysis and condition comparison analysis were performed using IBM SPSS Statistics 27 and survival analysis was performed using GraphPad Prism 9.1.2. Results are reported as mean ± SD. Normality was tested using the Shapiro–Wilk test. PA level as well as the percent of nocturnal time spent in different BG ranges (i.e., hypoglycemia, euglycemia (3.9-10.0 mmol/L), and hyperglycemia (>10.0mmol/L)), as measured by CGM, were compared between “ACT” and “L-ACT” conditions using either paired t-tests or the Wilcoxon matched-pair test. McNemar’s chi-squared test was used to compare two proportions and to compare the number of participants experiencing hypoglycemic events as well as the number of participants reporting additional CHO consumption. Effect of condition was assessed using a linear model.

Significant interactions were followed up with Bonferroni adjusted *post-hoc* tests. Spearman’s rank correlations were performed to analyze possible associations between PA level/duration of PA and times in, below and above range as well as hypoglycemia duration and mean change in glucose levels from midnight to 6:00 am. Statistical significance was set to *P<0.05*.

## Results

A total of 58 participants were recruited. After data cleaning procedures, data from 25 adults (10 males, 15 females) were analyzed (**Figure 1**). Baseline characteristics of study participants are presented in **Table 1**. Fourty-four percent of participants were treated with open-loop CSII and 56.0% were treated with MDI. No participant reported a significant macro- or microvascular event prior to the study. Impaired awareness of hypoglycemia was identified in three participants (12%) out of 25 by the Clarke questionnaire. Four participants (16%) had undetermined hypoglycemia awareness, 14 (56%) had normal hypoglycemia awareness, and four (16%) did not answer the questionnaire. We made sure that the conditions between ACT and L-ACT were significantly different in terms of PA level, total energy expenditure, and time spent in light, moderate, and vigorous-intensity PA (**Table 2**).

**Table 1:** Baseline characteristics of study participants.

N, total	25
**Insulin therapy** (CSII/MDI)	11/14
**Age, years**	34.8 ± 12.3 (20 – 60)
**Sex (Male/Female)**	10/15
**Ethnicity (Caucasian/Arab)**	21/4
**BMI, kg/m²**	25.4 ± 3.9 (20.7 - 34.7)
**Glycated hemoglobin (HbA_1c_), %**	7.6 ± 0.7 (5.9 - 8.7)
**Glycated hemoglobin (HbA1_c_), mmol/mol**	60 (41 – 72)
**Diabetes duration (years)**	16.8 ± 9.7
**Daily basal or long-acting insulin (u/day)**	20.8 ± 7.3 (11 – 36.5)
**VO_2peak_ (mL/kg/min)**	32.3 ± 9.3
**Daily PA level**	1.7 ± 0.2 (1.2 – 2.0)

Data are mean ± SD (min – max) or n (%).SD, standard deviation; CSII, Continuous subcutaneous insulin infusion; MDI, multiple daily injections; VO_2peak_; peak oxygen consumption.

**Table 2:** The title needs to be updated to: Comparison of accelerometry data between ACT and L-ACT.

	ACT	L-ACT			*P-value*
**PA level**	2.0 ± 0.3 (1.7 – 3.3)	1.4 ± 0.1 (1.1 – 1.6)			*<0.001*
**Light intensity (min)**	312.8 ± 109.0 (183 – 668)	247.4 ± 102.3 (71 – 462)			*0.018*
**Moderate intensity (min)**	172.7 ± 68.8 (66 – 287)	47.0 ± 31.4 (0 – 113)			*<0.001*
**Vigorous intensity (min)**	43.8 ± 56.5 (0 – 287)	1.4 ± 2.5 (0 – 11)			*<0.001*
**Sedentary (min)**	864.2 ± 143.4 (556 – 1124)	1096.7 ± 126.3 (835 – 1345)			*<0.001*
**Total energy expenditure (kcal)**	3195.6 ± 986.4 (2172 – 6498)	2262.2 ± 413.3 (1590 – 3325)			*<0.001*

ACT, Active day; L-ACT, less active day. Data are Mean ± SD (min-max).

### Nocturnal glucose profiles

Glucose values at bedtime were comparable in both conditions (P=0.250). We found no differences in the nocturnal time spent in level 1 hypoglycemia (3.0 – 3.9 mmol/L) nor level 2 hypogylcemia (<3.0 mmol/L) between conditions. Time spent below range (<3.9 mmol/L) was associated with greater nocturnal glycemic variation the night following ACT (CV) (R=0.648; P<0.001).

**Figure 2:**
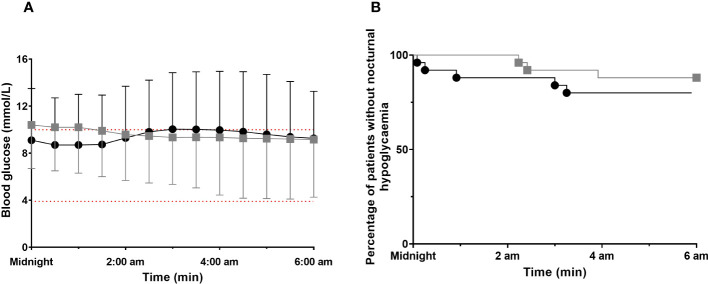
Nocturnal glucose profiles for both conditions (ACT vs. L-ACT) ACT, black circle; L-ACT, gray squares. **(A)** Glucose profiles from midnight to 6:00 am for both conditions. Values are means ± SD, Main effects by linear model time (P<0.001); condition ACT vs. L-ACT (P<0.001) and interaction (time x group) (P<0.001). **(B)** Time to first nocturnal hypoglycemic event.

We found a significant interaction (Condition ‘*ACT* vs *‘L-ACT’* × Time) reflecting a slight decrease in glucose levels in the first part of the night following L-ACT while glucose levels tended to increase during the second part of the night following ACT. However, there were no pairwise differences between both conditions in *post-hoc* analyses (**Figure 2**). Accordingly, data showed a greater decrease in glucose levels from midnight to 6:00 am the night following L-ACT (P<0.001) (**Table 3**).

**Table 3:** Nocturnal glucose and nocturnal hypoglycemia outcomes based on intersitial glucose measurements.

	ACT	L-ACT	*P* value
Glucose (mmol/L)			
At midnight	9.1 ± 4.4	10.5 ± 4.04	0.250
At 6:00 am	9.0 ± 4.2	9.1 ± 4.8	0.929
Nadir	6.0 ± 2.9	7.6 ± 3.6	0.150
SD	2.0 ± 1.7	1.3 ± 0.8	0.093
CV (%)	21.2 ± 13.8	15.1 ± 9.4	0.118
**Delta glucose (mmol/L)**			
Δ Midnight to 6:00 am	0.1 ± 6.2	-1.2 ± 4.1	**>0.001**
Δ Midnight to nadir	-3.2 ± 3.5	-2.8 ± 2.9	0.725
**Glucose ranges from 00:00 to 6:00 am (%)**			
< 3.0 mmol/L (Level 2)	4.4 ± 11.0	1.8 ± 5.9	0.687
3.0 to 3.9 mmol/L (Level 1)	3.4 ± 8.1	3.1 ± 9.1	0.804
< 3.9 mmol/L	7.5 ± 16.9	4.9 ± 14.2	0.957
3.9 to 10.0 mmol/L	56.1 ± 37.9	60.3 ± 40.2	0.825
> 10.0 mmol/L	36.3 ± 38.9	34.9 ± 42.0	0.856
> 13.3 mmol/L	15.8 ± 27.0	13.4 ± 32.4	0.801
**Hypoglycemia**			
NH (n)	5	3	0.702
Hypoglycemia duration (min)	139.6 ± 68.7	136.3 ± 70.1	0.950
Level 1 hypoglycemia duration (min)	59.4 ± 39.6	83.3 ± 35.3	0.650
Level 2 hypoglycemia duration (min)	80.2 ± 55.1	53.7 ± 40.2	0.500
Time to first hypoglycemia	115.2 ± 77.5	205.0 ± 52.0	0.130

Data are presented as mean ± standard deviation except for NH, reported as the number of subjects. *Significant difference between conditions. TIR, time in range; SD, standard deviation; CV, coefficient of variation; NH, Nocturnal hypoglycemia.

Based on interstitial glucose concentrations, hypoglycemia occurred in five participants (20%) during ACT night, three participants (12%) during L-ACT night, while 16 participants did not experience hypoglycemia on either night (64%). No participant experienced nocturnal hypoglycemia on both ACT and L-ACT nights. Nocturnal hypoglycemia occurred at similar times during the night in both conditions (P=0.130) (**Figure 2B**). Four out five nocturnal hypoglycemia events that occurred on ACT night were resolved at 6:00 am. Blood glucose increased to reach levels of 14.9 ± 7.2 mmol/L (min, 4.9 mmol/L; max, 20.5 mmol/L) following nocturnal hypoglycemia resolution. Only one out of three nocturnal hypoglycemia events on L-ACT night was resolved at 6:00 am. Blood glucose increased to reach levels of 4.9 mmol/L while the other two remained in nocturnal hypoglycemia, with one value below 3.0 mmol/L.

Time spent above range (BG > 10 mmol/L) during ACT and L-ACT is reported in **Table 3**, and was observed in more than half of the participants without any difference between conditions (60% of participants, ACT vs. 64%, L-ACT). Time spent in level 2 hyperglycemia (BG>13.9 mmol/L) was comparable as well (40% of participants, ACT vs. 25%, L-ACT; P=0.217).

The physical activity level on ACT Day was associated with a greater difference in nocturnal glucose levels from midnight to morning (R=0.664; P<0.01) as well as with a greater difference in nocturnal glucose levels from midnight to nadir (R=0.461; P=0.020). No other significant correlations between physical activity level on ACT day and hypoglycemia, euglycemia or hyperglycemia were detected.

### Self-reported PA on ACT Day

Sixteen out of twenty-five participants (64%) reported exercising (or leisure PA) on ACT Day. PA included biking, walking, running, swimming, high-intensity interval training, resistance training, skiing, and more. Seven participants (28%) reported exercising in the afternoon, three (12%) in the morning and six (24%) reported exercising twice: once in the morning and once in the afternoon. Nocturnal hypoglycemia occurred in two participants who reported performing PA twice during the day and in two participants who reported performing PA in the morning. Two nocturnal hypoglycemia events occurred in participants who had not reported PA in their logbook.

### Self-reported insulin- or CHO-based strategies the evening following ACT

Almost half of the participants (n=11; 44%) reported applying one, or a combination of insulin- and CHO-based strategies to reduce the risk of nocturnal hypoglycemia the night following ACT. Consumption of evening snacks (with or without insulin bolus) was the most frequently reported strategy (40%) (**Table 4**). CHO intake varied from 12g to 40g (mean 25.6 ± 13.3g) (**Table 5**).

**Table 4:** Participants reporting mitigation strategies to reduce the risk of nocturnal hypoglycemia.

	ACT		L-ACT	
Reported mitigation strategies
*Participants applying a strategy, n (%)*	CSII	MDI	CSII	MDI
**Evening snack**				
-With insulin bolus	3 (27.3)	0 (0)	0 (0)	0 (0)
-Without insulin bolus	2 (18.2)	5 (35.7)	1 (9)	5 (35.7)
**Basal insulin reduction**	2 (18.2)	0 (0)	0 (0)	0 (0)
**Evening snack with basal insulin increase (5%)**	1 (9)	0 (0)	0 (0)	0 (0)
**Evening meal bolus reduction**	0 (0)	0 (0)	0 (0)	0 (0)
**Evening insulin bolus correction**	3 (27.3)	4 (28.6)	6 (54.5)	2 (14.3)

One participant combined evening snack without insulin bolus and insulin basal rate increase, followed by insulin basal rate reduction during the night.

**Table 5:** Reported additional CHO and insulin bolus intakes during the evening.

	ACT	L-ACT	P value
Participants consuming additional CHO (post-meal), (n)	10	5	0.217
**Participants consuming CHO to treat hypoglycemia** - **In the evening (before midnight)** - **After midnight**	5 2	1 0	0.192 0.470
**Mean CHO consumption, (g)** - **CHO consumed to avoid hypoglycemia** - **CHO consumed to treat hypoglycemia** - **Total CHO consumed**	25.6 ± 13.3 (12 – 47) 30.0 ± 20.0 (20 – 60) 27.7 ± 15.6 (12 – 60)	19.5 ± 11.0 (9.7 – 33) (-)** 19.5 ± 11.0 (9.7 – 33)	0.399 - 0.045*
**Participants administrating insulin bolus corrections, (n)**	7	5	
**Mean insulin bolus corrections, (u)**	2.3 ± 0.7 (1.5 – 13.7)	1.9 ± 0.7 (1.1 – 2.5)	0.074

Data are Mean ± SD (min – max) or number of participants (n). *Significant difference between conditions “ACT” vs. “L-ACT” (P<0.05). One participant reported treating hypoglycemia during the evening on L-ACT day without specifying means of correction. **One participant reported CHO intake to treat hypoglycemia during the evening (before midnight) but did not specify quantity or quality of CHO consumed.

In participants treated with CSII, only one reported using temporary insulin basal rate reduction (BRR) as a mitigation strategy for nocturnal hypoglycemia which consisted of a 50% BRR for 4-hours. One participant reported increasing the basal rate by 5% until 1 am after consuming a snack without insulin bolus.

### Hypoglycemia on the evening and night following ACT

Reported hypoglycemia during the evening following ACT was always treated with CHO. One participant reported a 50% BRR for 3-h during the night in addition to CHO intake. The type or quantity of CHO consumed to treat hypoglycemia was not always clearly reported.

Five participants reported treating hypoglycemia during ACT evening (before midnight), and did not experience subsequent hypoglycemia between midnight and 6:00 am. Two participants reported treating symptomatic hypoglycemia just after midnight and did not experience subsequent nocturnal hypoglycemia either, during midnight and 6:00 am. Based on interstitial glucose values, both participants did not experience what was considered as significant hypoglycemia. One had blood glucose levels > 3.9 mmol/L but reported symptoms, and the other had blood glucose levels <3.9 mmol/L for less than 15 minutes. None of the five participants who experienced significant nocturnal hypoglycemia reported it in their logbook.

Among the participants who experienced nocturnal hypoglycemia the night following ACT, two had administrated additional insulin boluses during the evening (3 and 1.5 u); two had an evening snack (one with, and one without insulin bolus); one had an evening snack with an insulin basal rate increase of 5% and one did not report applying any mitigation strategies or insulin bolus injections.

### Self-reported insulin- or CHO-based strategies the evening and night following L-ACT

During the evening following L-ACT, six participants (24%) reported eating an evening snack (≈ 19.5 ± 11.0g CHO). Seven (28%) reported administrating an insulin bolus correction throughout the evening without extra-CHO consumption (mean insulin units: 1.9 ± 0.7).

### Hypoglycemia on the evening and night following L-ACT

One participant reported correcting hypoglycemia in the evening without specifying symptoms or means of correction, but did not experience subsequent nocturnal hypoglycemia. Out of the three participants who had nocturnal hypoglycemia the night following L-ACT, only one reported administrating an insulin bolus correction in the evening.

### CHO intake the evening following ACT vs. L-ACT

Post-meal CHO was consumed in greater quantities in the evening following ACT (P=0.045). Additional insulin bolus injections tended to be higher in the evening following ACT (P=0.074) (**Table 5**).

## Discussion

Most studies have tested the impact of PA on glycemic excursions under standardized conditions. This work aimed to examine the association between free-living daily physical activity level and nocturnal blood glucose levels in people living with T1D while taking into account possible mitigation strategies reported by participants to avoid nocturnal hypoglycemia. Our results suggest that nocturnal hypoglycemia occurrence and time spent below range are no different the night following an active day versus the night following a less active day, which is likely due to higher carbohydrate intake the evening following ACT day.

### Glycemic excursions

It is well known that nocturnal hypoglycemia often occurs in patients with T1D during or up to 31-h following PA (10). Obviously, being active during the day may come at a cost. Previous studies (10–12) have reported that time spent below range tended to increase for several hours following PA. In a recent survey-based study, 49% of people living with T1D reported experiencing nocturnal hypoglycemia following PA (48). In the current study, we found that only 20% of participants experienced nocturnal hypoglycemia the night following ACT day and found no differences in nocturnal hypoglycemia occurrences the night following ACT day versus the night following L-ACT day. Five participants reported correcting hypoglycemia in the evening on ACT day (before midnight) and, two others just after midnight. These were not the same participants who experienced nocturnal hypoglycemia later during the night. This could be an explanation as to why we found no differences in the number of nocturnal hypoglycemia events between ACT day and L-ACT day. Hypoglycemia associated with daily PA may have occurred earlier in the evening.

Times in range (3.9 – 10.0 mmol/L) and below range (<3.9 mmol/L) were comparable the night following an active day versus the night following a less active day. Riddell et al. reported that participants spent more time in range on active days than on less active days, but also more time below range (49). Authors reported increased time below range in the first 12 hours following PA. This disparity might be partly explained by the fact that in the latter study, participants performed structured PA, whereas, in the current study, the chosen distribution is closer to free-living conditions (i.e., uncontrolled PA with variable duration and modality).

In contrast, we found that over 60% of participants actually experienced hyperglycemia the night following ACT day, including participants who had experienced nocturnal hypoglycemia earlier in the night (3 out of 5). This suggests that people may have treated hypoglycemia without reporting it and might also explain why glucose levels increased the night following ACT day while they slightly decreased the night following L-ACT day. More importantly, people living with T1D have frequently reported overeating following hypoglycemia and trying to compensate high-risk situations by maintaining higher BG levels (50). Nighttime constitutes a critical period of the day, leaving people with T1D at higher risk of severe hypoglycemia which could result in seizures or even death (19, 51). This could explain why more than half of the participants spent time above range at night, regardless of their PA the previous day. This suggests that high nocturnal blood glucose levels may be related to participants aiming for higher glucose levels at night to avoid nocturnal hypoglycemia and not to additional CHO intake due to PA.

Our study looks at the percentage of time in glycemic range in people treated with open-loop CSII or MDI. We found that time in range was 56.1 ± 37.9% from 00:00 to 6:00 am the night following ACT. Newer technologies, such as hybrid closed-loop systems are frequently associated with higher time in range (3.9 – 10.0 mmol.L) during and in the hours following exercise. Breton et al. (52) tested closed-loop systems during and after intense prolonged exercise in adolescents with T1D. Authors found higher time in range with closed-loop systems, compared to standard CSII, especially late at night. Time below range remained similar in both conditions. In line with these results, Tauschmann et al. (53) found that hybrid closed-loop therapy was associated with higher time in range and lower time above range compared to CSII, in free-living conditions in adolescents with T1D. Authors found no difference in time below range between CSII and hybrid closed-loop. Thus, including hybrid closed-loop systems in the current study may have resulted in higher time in range without having an impact on time below range.

### Self-reported insulin- or CHO-based strategies

A limited number of studies evaluating strategies used by people with T1D to manage PA-induced glucose variability have been published. Our results showed that only 44% of participants reported applying a strategy in order to reduce the risk of nocturnal hypoglycemia associated with PA. As ACT days were based on mean daily PA level, what was calculated as PA by the accelerometer, was not always reported as PA by the participant. Therefore, habitual PA or a cumulation of activities of daily-living (fast walks, fast walking up the stairs, etc.) throughout the day may have led to a mean physical activity level >1.7. Thus, it may be more difficult for people with T1D to identify their activities as PA and adjust their treatment in consequence. This could be a possible explanation as to why 56% of our participants did not report applying a compensation strategy.

Eating snacks with or without insulin bolus seemed to be the most recurrent strategy used in the evening following ACT day in the current study. This was confirmed by Pinsker et al. (54) in a survey-based study aiming to examine strategies for PA preparation in people living with T1D. Authors reported that most people would consume supplementary CHO to avoid hypoglycemia during and in the hours following PA, regardless of insulin therapy (CSII or MDI) or CGM use (54). In terms of prevention strategies for PA-associated hypoglycemia, carbohydrate feeding often requires less pre-planning compared to basal and bolus adjustments and therefore, may be more common than strategies based on insulin reduction.

We also focused on the impact of treatment (e.g., CSII vs. MDI) on the decisions taken by people with T1D to manage their blood glucose in the hours following PA in free-living conditions. In the current study, two (18.2%) participants reported reducing their basal insulin in the evening following PA. One of them experienced nocturnal hypoglycemia. Paiement et al. (48) showed that among CSII users, those applying insulin BRR during the night following PA reported more nocturnal hypoglycemia (48).

Pinsker et al. (54) found that most people using BRR strategies were those treated with combined CSII and CGM. In our study, participants’ CGMs were blinded which could explain why only two participants applied insulin basal rate reduction to prevent hypoglycemia. One participant reported reducing their basal rate to treat hypoglycemia. The authors also found that insulin bolus reduction for the meals around PA was reported by half of the participants (54). No meal insulin bolus reduction was reported in our study. In Groat’s (55) survey-based study, no participant reported reducing their insulin meal boluses.

Most of the participants (three out of five) who experienced nocturnal hypoglycemia had reported applying a mitigation strategy. However, some participants either increased their insulin basal rate during the evening or administrated an additional insulin bolus on multiple occasions after CHO intake. This may be the cause of nocturnal hypoglycemia, rather than late-onset effects of PA. Indeed, Desjardins et al. (2) reported that CHO supplementation resulted in higher nocturnal hypoglycemia occurrences when associated with insulin injection. Two others who experienced nocturnal hypoglycemia administrated an additional insulin bolus correction in the evening (9:45 pm – 10:00 pm) nonrelated to CHO supplementation. In the hours following PA, insulin sensitivity is increased, and muscle and hepatic glycogen content need to be restored which results in glucose being diverted from the blood, increasing the risk of hypoglycemia (56). Thus, enhanced insulin sensitivity associated with an additional insulin bolus injection in the evening would increase the risk of hypoglycemia even further.

### Strengths and limitations

Overall, the strength of this observational study is to assess life habits with a focus on daily physical activity and glucose control over two days (one considered as active, the other as less-active) in non-standardized conditions. Besides, based on participants’ reports, CGMs, and accelerometry data, we were able to assess whether mean daily physical activity level has an impact on nocturnal glucose fluctuations and whether people living with T1D use compensation strategies to reduce the risk of nocturnal hypoglycemia associated with PA. Literature evaluating strategies to reduce the risk of late-onset and post-PA hypoglycemia is scarce and non-standardized studies evaluating these strategies are even less frequent. Our study helps to identify a potential lack of knowledge in terms of post-PA mitigation strategies in people living with T1D.

An important part of our study relied on participants recalling information correctly. Information regarding dietary intakes (quantity and quality) and insulin adjustments as mitigation preventive strategies, as well as nocturnal hypoglycemia or nocturnal hypoglycemia correction, was sometimes incomplete or not reported. There may also have been discrepancies or inaccuracies in the self-reported data, such as omission of insulin bolus corrections or hypoglycemia correction. Our analysis relied on a small sample-size which may have resulted in a lack of statistical power.

## Conclusions

In conclusion, nocturnal hypoglycemia does not seem to appear more frequently on nights following an active day. Post-meal carbohydrate intake was significantly higher on evenings following an active day, indicating compensation strategies to avoid nocturnal hypoglycemia. Nocturnal hypoglycemia occurred more frequently in participants who administrated insulin bolus corrections in the evening with or without extra carbohydrate consumption. These results suggest that, although people with T1D seem to be aware of the increased risk of nocturnal hypoglycemia associated with PA, the risk associated with additional insulin boluses may not be as clear. Most participants did not report using compensation strategies to reduce the risk of late-onset hypoglycemia associated with PA which may be because they did not consider habitual PA as something requiring treatment adjustments.

## Data availability statement

The original contributions presented in the study are included in the article/supplementary material. Further inquiries can be directed to the corresponding authors.

## Ethics statement

The studies involving human participants were reviewed and approved by IRCM ethics committee. The patients/participants provided their written informed consent to participate in this study.

## Author contributions

JM, RR-L, EM-C and ST contributed to the conception and design of the study. CS and VM coordinated the study and acquired the data. JM and ST analyzed the data. JM, EM-C, RR-L and ST interpreted the data. JM and ST drafted the manuscript. JM, ST, EM-C, CS, VM, KP, EH, RR-L critically revised the manuscript for important intellectual content. All authors approved the final version of this manuscript.

## Funding

This study was supported by funds the J.-A. DeSève Fondation diabetes chair held by RR-L. ST was supported by grants from the Société Francophone du Diabète- Grant number gm/cc21–01 Fondation pour la Recherche Médicale and Cardiometabolic Health, Diabetes and Obesity Research Network. ST, EH, VM, and RRL are members of the International Associate Laboratory REGALE-1 (Glycaemic regulation during exercise in type 1 diabetes) labeled by Lille university in 2020.

## Acknowledgments

We are thankful to all the participants who dedicated their time and efforts to completing this study. We would like to equally acknowledge the contributions of the diabetes nurses at Montreal Clinical Research Institute to the conduction of the interventions.

## Conflict of interest

Research grants: Diabetes Canada, Astra-Zeneca, E Lilly, Cystic Fibrosis Canada, CIHR, FFRD, Janssen, JDRF, Merck, NIH, Novo-Nordisk, Société Francophonedu Diabète, Sanofi-Aventis, Vertex Pharmaceutical. Consulting /advisory panel: Abbott, Astra-Zeneca, Bayer, Boehringer I, Dexcom, E Lilly, HLS therapeutics, INESSS, Insulet, Janssen, Medtronic, Merck, Novo-Nordisk, Pfizer, Sanofi-Aventis. Honoraria for conferences: Abbott, Astra-Zeneca, Boehringer I, CPD Network, Dexcom, CMS Canadian Medical&Surgical Knowledge Translation Research group, E Lilly, Janssen, Medtronic, Merck, Novo-Nordisk, Sanofi-Aventis, Tandem, Vertex Pharmaceutical. Consumable gift (in Kind): E Lilly, Medtronic Unrestricted grants for clinical and educational activities: Abbott, E Lilly, Medtronic, Merck, Novo Nordisk, Sanofi-Aventis Patent: T2D risk biomarkers, catheter life Purchase fees: E Lilly (artificial pancreas).

## Publisher’s note

All claims expressed in this article are solely those of the authors and do not necessarily represent those of their affiliated organizations, or those of the publisher, the editors and the reviewers. Any product that may be evaluated in this article, or claim that may be made by its manufacturer, is not guaranteed or endorsed by the publisher.
